# Obituary: Prof. István M. Ábrahám

**DOI:** 10.3389/fendo.2023.1144417

**Published:** 2023-06-14

**Authors:** Zsuzsanna Nagy, Klaudia Barabas, Gergely Kovacs

**Affiliations:** Institute of Physiology, Medical School, University of Pécs, Pécs, Hungary

**Keywords:** estrogen, neurodegenaration, fertility, classical way, non-classical way

The authors dedicate this special issue in Frontiers in Endocrinology to Prof. István Ábrahám, on the occasion of his passing away in April 2021.

Prof. Dr. István Miklós Ábrahám

(1967-2021)

István Ábrahám graduated *summa cum laude* from the University Medical School Pécs, in 1993. As a student, he began research at the Institute of Physiology, Neurophysiology Research Group of the Hungarian Academy of Sciences, under the guidance of Professor László Lénárd. During his undergraduate years, Professor Ábrahám achieved outstanding results for which he was awarded the Fellowship of the Republic in sequentially three times. Additionally, he was awarded a Demonstrator Fellowship in the Department of Physiology, and in 1993, he was awarded the prestigious Pro Scientia Gold Medal.

Uniquely, in 1993, he presented two lectures at the National Conference of the Undergraduate Research Society, for which he received one First and one Second Prize.

Following graduation, he continued his PhD studies at the Institute of Experimental Medicine in Budapest, under the supervision of Dr. Krisztina Kovács. During this time, he broadened his professional knowledge in the research group of world-renowned neuroendocrinologist Béla Bohus, at the University of Groningen in the Netherlands. He defended his PhD thesis *summa cum laude* at the School of PhD Studies, Semmelweis University of Medicine in Budapest in 1998.

After earning his PhD, he spent two more years at the Molecular Neuroendocrinology Research Group of the Institute of Experimental Medicine, where his research focused on stress-related neuronal networks.

Between 2000 and 2002, he was a Marie Curie Fellow in Prof. Allan Herbison’s laboratory at the Babraham Institute in Cambridge, England, where he developed a lifelong professional relationship with Professors Allan Herbison and Seong Kyu Han. He studied the concentration dependent action of glucocorticoids on neuronal cell viability and cell survival in the brain. His interest then shifted towards studying the non-genomic effects of estrogen in the brain.

Following his return home, he became one of the leading researchers in the Neurobiology Research Group of the Hungarian Academy of Sciences at the Eötvös Loránd University (Budapest) for a 4-year period, in which he continued studying the effects of estrogen in the brain. During this time, two PhD students obtained their doctoral degrees under his professional supervision.

In 2007, he was offered the opportunity to set up and manage his own research group at the University of Otago in New Zealand, where he achieved considerable professional success. During the six years he spent in New Zealand, two other students completed their PhD studies under his guidance. While in Otago, he developed a close collaboration with Professor Akihiro Kusumi in the field of single molecule detection. It was this collaboration which gradually shifted his interest towards super-resolution microscopy.

Despite his success abroad, his heart always remained in Hungary, where he envisioned a future for his children and his family. Eventually, he returned to his Alma Mater in 2011, where he started to work in part time.

With the support of the Albert Szent-Györgyi Scholarship, among others, Professor Ábrahám began implementing his innovative ideas in 2013. Following his appointment as Professor at the Department of Phyiology, he founded the Molecular Neuroendocrinology Research Group, which has consistently undergone expansion, and evolved into a professionally diverse and exceptionally cohesive group in the following years. In 2013, an academic research doctorate was also awarded to him.

He was instrumental in founding and chairing the first Centre for Neuroscience in the country, at the University of Pécs. Professor Ábrahám served on several editorial boards of international scientific journals and scientific societies. In early 2021, he was elected President of the Hungarian Neuroscience Society.

Following his appointment as Director of the Institute of Physiology in 2019, István immersed himself into the task of reforming the institute with his characteristic drive and determination. Additionally, he exerted immense energy in seeing one of his greatest dreams take flight, which was the creation of a facility accommodating a wide range of super-resolution and advanced fluorescence microscopes. In Spring 2021, the equipment was about to be set up at its new premises, designed by Professor Árbahám; but tragically, he never saw this completed. The centre was launched at its final location and named István Ábrahám Nano-Bio-Imaging Core Facility in December 2021.

István had an excellent scientific carrier with many fruitful professional collaborations. Besides being an outstanding scientist, he was an excellent leader, a great teacher, and a very good friend. We all miss him.

## Author contributions

All authors listed have made a substantial, direct, and intellectual contribution to the work and approved it for publication.

